# Beam Diameter Dependence of Performance in Thick-Layer and High-Power Selective Laser Melting of Ti-6Al-4V

**DOI:** 10.3390/ma11071237

**Published:** 2018-07-18

**Authors:** Wentian Shi, Yude Liu, Xuezhi Shi, Yanjun Hou, Peng Wang, Guohua Song

**Affiliations:** 1 School of Materials Science and Mechanical Engineering, Beijing Technology and Business University, Beijing 100048, China; shiwt@th.btbu.edu.cn (W.S.); liuyd@th.btbu.edu.cn (Y.L.); houyanjun@sina.com (Y.H.); wangpeng_12321@126.com (P.W.); 2 School of Naval Architecture and Mechanical-electrical Engineering, Zhejiang Ocean University, Zhoushan 316022, China; 3 Beijing Xinghang Mechanical and Electrical Equipment Co. Ltd., Beijing 100074, China; sghbit@sina.com

**Keywords:** selective laser melting, high layer thickness, high power, beam diameter, Ti6Al4V

## Abstract

A 400 W high-power laser was used to fabricate 200-µm-thick Ti-6Al-4V samples to evaluate the effects of small (50 μm) and large (200 μm) beam diameter on density, microstructure and mechanical properties. A series of single-track experiments demonstrated that it was challenging for the small-beam laser to fabricate smooth and defect-free scan tracks. A larger beam diameter efficiently avoided process instability and provided a more stable and uniform melt pool. By increasing the beam diameter, the density of multilayer samples reached 99.95% of the theoretical value, which is much higher than that achieved with the small beam diameter. However, it was difficult to completely eliminate defects due to serious spatter and evaporation. Moreover, all of the generated samples had relatively coarse surfaces. For the large beam diameter of 200 µm, the optimal yield strength, ultimate tensile strength and elongation were 1150 MPa, 1200 MPa and 8.02%, respectively. In comparison, the small beam diameter of 50 µm resulted in values of 1035 MPa, 1100 MPa and 5.91%, respectively. Overall, the large-diameter laser is more suitable for high-power selective laser melting (SLM) technology, especially for thick layers.

## 1. Introduction

Selective laser melting (SLM) is an additive manufacturing technique that selectively melts successive layers of metal powder using a laser beam [1,2,3,4]. SLM is the most promising additive manufacturing technology because it can generate simple or complex metal parts with low surface roughness, high density and excellent mechanical properties [5,6]. Currently, during the SLM processing, thin layer thicknesses of 20–50 μm and a small beam having a focused spot size ranging from 30 to 100 μm are used to guarantee high surface precision but limit the forming efficiency [7,8,9,10,11,12]. Undoubtedly, lower forming efficiency is the main bottleneck in SLM technology, which is strongly associated with scanning speed, layer thickness and hatch spacing. Scanning speed and layer thickness are limited by the available laser power, while hatch spacing is linked to the width of the melt track and is typically about 0.7-times the track width [13].

Recent studies have demonstrated that increasing the laser power enables higher scanning speeds and the use of greater layer thicknesses, which improves forming efficiency [14,15]. In general, a thin layer thickness ensures precise SLM samples. As the layer thickness continues to increase, a higher energy input is required to melt the thicker powder layer, and this large energy input can easily lead to splashing and defects. For example, Ma et al. applied a high-power SLM laser to various thicknesses of stainless steel. When the layer thickness exceeded 100 μm, the penetration depth exceeded 750 μm and voids were formed at the bottom of the melt channel [16]. This is because, with increasing laser power at constant beam diameter, the beam intensity increases and a keyhole is easily generated, resulting in melt splashing and evaporation phenomena. To increase the forming efficiency and avoid process instability, personnel from the Fraunhofer Institute of Laser Technology modified an SLM machine to equip it with a 1000 W laser source and a redesigned optical system that allows changing the beam diameter between 200 and 1000 µm. Schleifenbaum et al. prepared a high-density aluminum sample using a 500 W laser with a spot diameter of 800 μm [14]. Buchbinder et al. used a 900 W laser with a spot diameter of 1000 μm for the production of AlSi10Mg parts and reported an increase in forming efficiency from 4 to 21 mm^3^/s. These studies indicate that increasing the beam diameter could avoid instability in the high-power laser SLM process [17]. However, the authors believed that low thermal conductivity metals, such as Ti-based alloys, could not be processed at high laser powers while maintaining a constant beam diameter [3].

Additively manufactured Ti-6Al-4V has been widely studied due to its superplasticity, low weight and high mechanical strength [4,18,19,20,21]. We recently reported using a relatively large laser beam of diameter 200 μm to successfully avoid process instability in high-power SLM of Ti-6Al-4V with a high layer thickness of 200 µm [10]. On this basis, a thorough investigation into the effect of laser beam diameter on formability is warranted. Few publications have detailed the effect of beam diameter variation in high-power SLM. This article contrasts the effect on forming of small (50 μm) and large (200 μm) beam diameters. Herein, the influence of different beam diameters on density, microstructure and mechanical properties by high laser power SLM technology are reported. The mechanism of defect formation at different beam diameters and the variation in defect morphology are also analyzed. 

## 2. Experimental Procedures

### 2.1. Materials

**Figure 1:**
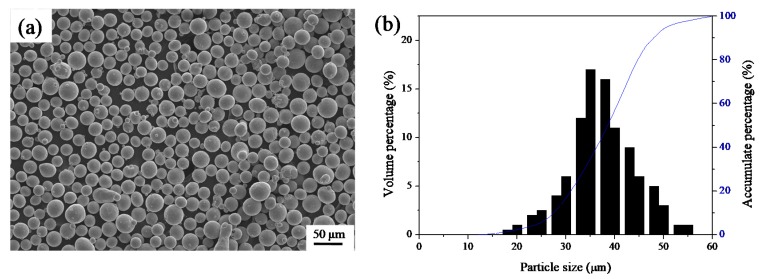
(**a**) Ti-6Al-4V powder morphology; (**b**) Particle size distribution.

The gas-atomized Ti-6Al-4V powder used in this study was obtained from Concept Laser GmbH (Franconia, Germany) and had a particle size ranging from 15 to 58 μm (d10: 22 μm; d50: 37 μm; d90: 48 μm). The morphology and particle size distribution of the powders are shown in Figure 1a,b, respectively. The chemical composition (see Table 1) corresponded to the ASTM F136-02a (ELI Grade 23) standard [18].

### 2.2. Experimental Setup and Manufacturing Process

**Figure 2:**
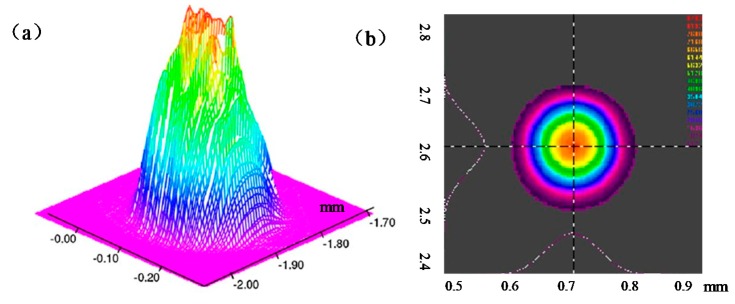
(**a**) Three-dimensional intensity distribution of the Gaussian beam; (**b**) Field distribution of the Gaussian beam in the focal plane.

SLM experiments were performed using a Concept Laser M2 Cusing machine (Concept Laser GmbH, Franconia, Germany). This SLM machine was equipped with a 400 W laser source and an optical system with a wavelength of 1075 nm in continuous wave mode that allowed the beam diameter to be varied between 50 and 200 µm. The laser source used within this setup was a fiber-coupled disk laser. The beam profile was Gaussian shaped. The flanks of the beam profile were steeper than those of a pure fundamental mode (Figure 2a) and the energy intensity changed with the position of the laser beam. The intensity was highest in the middle and then decreased steadily outwards (Figure 2b).

**Figure 3:**
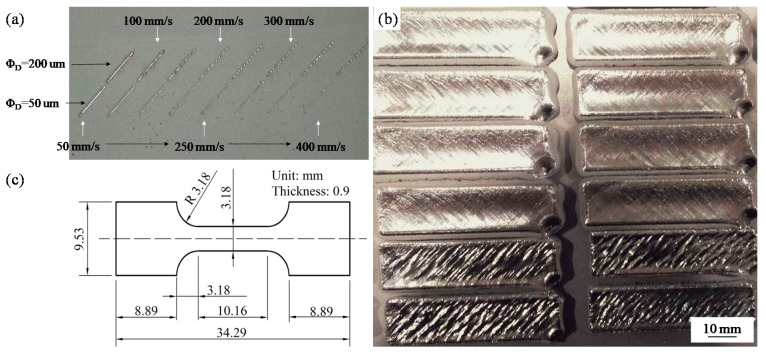
(**a**) Macrographs of single-scan tracks; (**b**) Macrographs of the Ti6-Al-4V samples; (**c**) Geometrical shape and size of a tensile test specimen.

In the experiments, the build chamber was backfilled with argon gas, used as a protective gas to maintain the oxygen concentration below 80 ppm. All of the samples were melted at a constant high laser power of 400 W and a layer thickness of 200 µm. To identify the range of suitable parameters for manufacturing the Ti-6Al-4V alloy, a series of single tracks, each with length 10 mm, were first melted. Two tracks were melted at different positions of the substrate in each condition to prevent uneven layer thickness. Single tracks were melted at two different beam diameters of 50 and 200 µm, and at laser scanning speeds ranging from 50 to 400 mm/s with a step of 50 mm/s (Figure 3a); these tracks were obscured and interrupted when the scanning speed was greater than 200 mm/s. Based on these results, the scanning speed was further divided, and the process window established. Then, Ti-6Al-4V specimens (Figure 3b) with dimensions of 40 mm × 10 mm × 4 mm (20 layers) were produced using the set of parameters chosen via a meander scanning strategy. The hatch spacing, which is the distance between consecutive scan tracks, was set at 0.7-times the track width.

### 2.3. Characterization

The surfaces of the single tracks were characterized by scanning electron microscopy (SEM) (JSM6490; JEOL, Tokyo, Japan) after fabrication. After evaluating the surface morphology, all of the samples were sectioned by a wire-cutting machine (Cmne; Beijing, China) and then ground with 200–2000 grit grinding papers, then polished with 1.5 and 0.5 µm polishing paste and finally polished with an oxide polishing suspension on a soft cloth. Metallographic specimens were prepared by standard mechanical polishing and etched with a solution of HF (2 mL), HNO_3_ (6 mL) and H_2_O (90 mL). The cross-sectional microstructures were observed using an optical microscope (DM4000M; Leica, Wetzlar, Germany) and SEM. The geometrical characteristics of the single tracks were examined by analyzing optical micrographs, which were quantified using ImageJ software (NIH, Bethesda, MD, USA). The relative density of the samples was measured by image analysis method. The porosity was examined using seamless stitching micrographs and quantified using ImageJ software. For each track and sample, two cross-sections at different locations were measured and averaged. The tensile test pieces were cut from the middle parts perpendicular to the building direction of the sample and then machined to the required dimensions (Figure 3c) by a wire-cutting machine. The tensile tests were examined using an Instron material testing machine (model 5966; Instron, Boston, MA, USA) to evaluate the tensile properties at room temperature. The crosshead displacement speed was 0.01 mm/s and a dynamic strain gauge extensometer was used to record the strain. The reported tensile properties were the average values for at least three specimens. For each condition, three samples were measured and averaged.

## 3. Results and Discussion

### 3.1. Single-Scan Tracks

During the SLM process, single-scan tracks are successively deposited one upon the other to finally form the sample. Hence, the properties of SLM parts strongly depend on the properties of each single track [13,22]. Furthermore, by evaluating the scan track characteristics, such as the surface morphology and geometrical features, significant information concerning the selection of process parameters can be gained. In this section, single tracks were melted using small (50 μm) and large (200 μm) beam diameters to assess the influence of beam diameter on the single-track geometrical features. The surface morphology and the cross-section geometrical characteristics of single tracks were investigated. Suitable process parameters were also selected.

#### 3.1.1. Surface Morphologies

**Figure 4:**
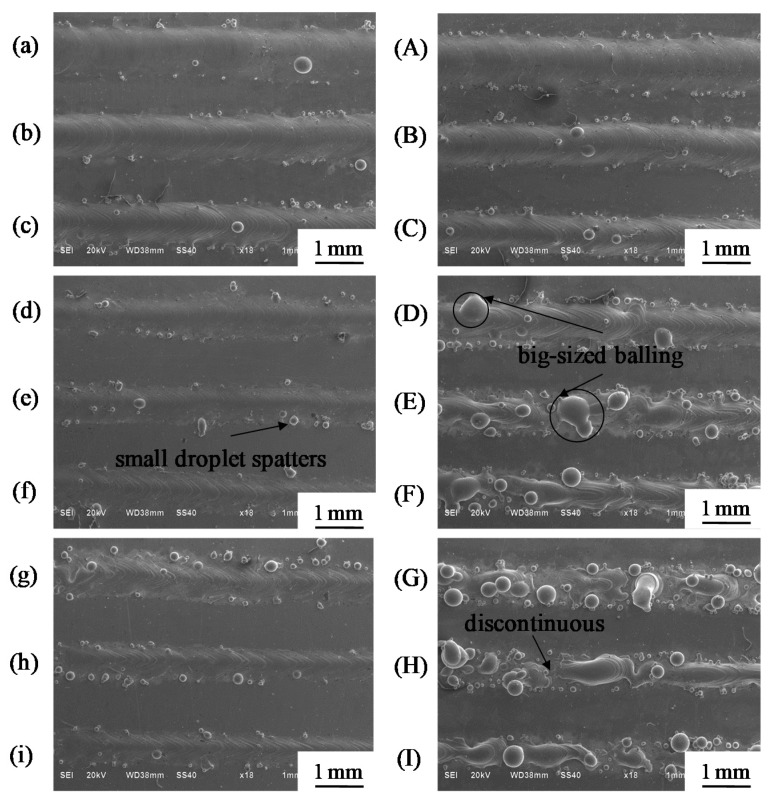
SEM micrographs showing the surface of single-scan tracks produced with different laser beam diameters (the beam diameter Φ_D_ is 50 µm in the left column and Φ_D_ is 200 μm in the right column). (**a**,**A**) v = 25 mm/s; (**b**,**B**) v = 50 mm/s; (**c**,**C**) v = 75 mm/s; (**d**,**D**) v = 100 mm/s; (**e**,**E**) v = 125 mm/s; (**f**,**F**) v = 150 mm/s; (**g**,**G**) v = 175 mm/s; (**h**,**H**) v = 200 mm/s; (**i**,**I**) v = 225 mm/s.

Figure 4 depicts the surface morphology of single-scan tracks under different beam diameters; the left column refers to the small beam diameter of 50 µm and the right column to the large beam diameter of 200 µm. From the vertical perspective, increasing scanning speed causes the scan track surface to change from smooth to rough; meanwhile the track width becomes increasingly narrow. This behavior was observed for both beam diameters. However, the large beam diameter was more sensitive to the scanning speed than the small beam diameter. With the large beam diameter, the scan tracks were continuous and provided a smooth surface at low scanning speeds ranging from 25 to 75 mm/s (Figure 4A–C). When the scanning speed exceeded 100 mm/s, balls began to appear (Figure 4D). As the speed increased, more large-sized particles appeared and resulted in the formation of discontinuous scan tracks (Figure 4D–I). This is because the energy density was insufficient to melt the powder at high scanning speed, thus causing serious balling [23]. However, at the small beam diameter of 50 μm, the scan tracks were continuous and small droplet spatters were apparent on the track surfaces at scanning speeds ranging from 25 to 225 mm/s (Figure 4a–i). Overall, with increasing scanning speed, the change in surface morphology of a single-scan track with the large beam diameter is more prominent than that with the small beam diameter. This is because, when all other process parameters are the same, the smaller the beam diameter, the larger the laser energy density, and thus with increasing speed, the energy input first appears insufficient with the large beam diameter.

#### 3.1.2. Cross-Sectional Profiles

The cross-sectional profile of each track was studied to better analyze and quantify the effect of beam diameter on the geometrical characteristics of the single tracks.

**Figure 5:**
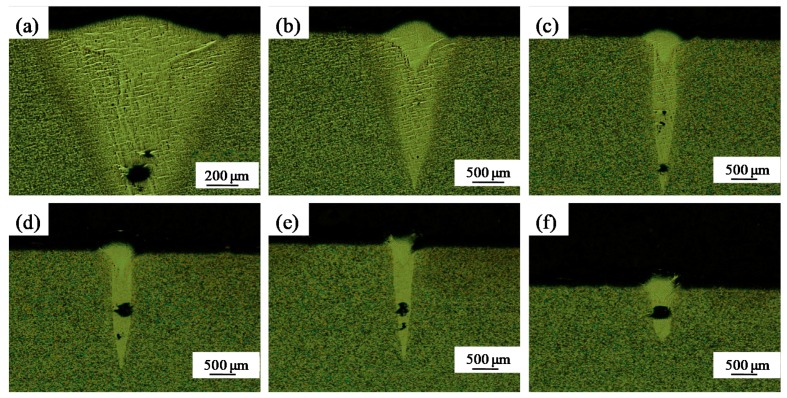
Optical micrographs showing the cross-sections of single-scan tracks produced with a small-diameter (50 µm) laser beam (P = 400 W, δ = 200 μm). (**a**) v = 25 mm/s; (**b**) v = 50 mm/s; (**c**) v = 75 mm/s; (**d**) v = 100 mm/s; (**e**) v = 150 mm/s; (**f**) v = 200 mm/s.

Figure 5 shows the morphology of the cross-sectional profiles at different scanning speeds when the beam diameter was 50 μm. The shape of the cross-section of single-scan tracks was funnel-shaped and each single track was deep (Figure 5). Several holes were evident on the bottom of each single track. This is because using high laser power while maintaining a small beam diameter increases the laser intensity. This in turn leads to a higher evaporation rate, resulting in a higher incidence of spattering, which is detrimental to the process as a whole [3]. Several small-sized liquid droplets splashed from the liquid front as it solidified.

**Figure 6:**
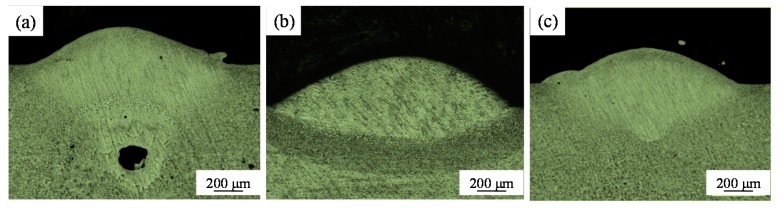

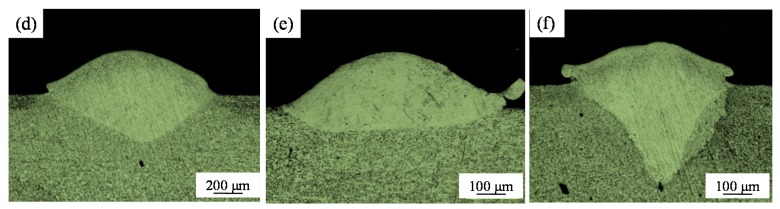
Optical micrographs showing the cross-sections of single-scan tracks produced with a large-diameter (200 µm) laser beam (P = 400 W, δ = 200 μm). (**a**) v = 25 mm/s; (**b**) v = 50 mm/s; (**c**) v = 75 mm/s; (**d**) v = 100 mm/s; (**e**) v = 150 mm/s; (**f**) v = 200 mm/s.

Figure 6 shows the morphology of the cross-sectional profiles at different speeds for a beam diameter of 200 μm. The penetration depth of the melt pool (Figure 6a–f) was much lower than that with the small beam diameter of 50 μm (Figure 5). However, when the speed was 25 mm/s, the penetration depth of the melt pool was still large and holes were produced (Figure 6a). With increased scanning speed, the penetration depth diminished, and the cross-sectional morphology of the melt pool became fully dense and elliptical (Figure 6b–d). This is because the laser energy was more dispersed rather than concentrated into the substrate or solidified layer below. This decreased the penetration depth and ensured good defect-free metallurgical bonding. However, at the highest scanning speeds the energy density was too low to fully melt the powder and substrate and the balling effect occurred; the wetting effect deteriorated, and large balls adhered to both sides of the track (Figure 6e,f).

#### 3.1.3. Geometrical Characteristics

**Figure 7:**
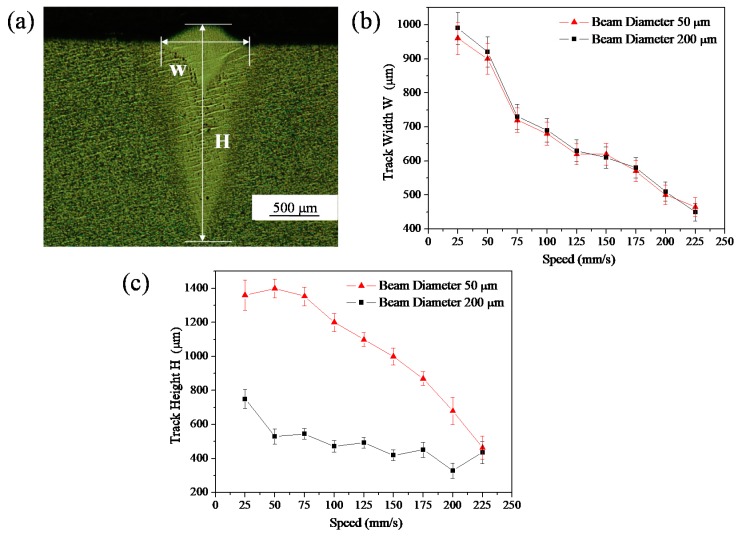
(**a**) Measurement of the cross-section of the scan track geometry; (**b**,**c**) Geometrical characteristics of the single tracks as a function of scanning speed for different beam diameters.

The geometrical characteristics (track width (W) and height (H)) of the cross-sections of single tracks were measured (Figure 7a). Figure 4b,c shows the relationship between the geometrical characteristics of synthesized single tracks and scanning speed for the different beam diameters. It is evident that the beam diameter had little effect on track width. At the same scanning speed, the track width was almost the same for the different beam diameters. Furthermore, the track narrowed from 1000–450 μm with increasing scanning speed (Figure 7b). However, the beam diameter strongly influenced the melt pool depth (Figure 7c). For the small beam diameter of 50 μm, the depth of the melt pool decreased from 1430–460 μm with increasing scanning speed. The variation in depth with scanning speed was less when the beam diameter was 200 μm, i.e., the depth decreased from 790–450 μm (Figure 5b). Generally, the cross-sectional characteristics of single tracks with the small beam diameter were more variable than those with the large beam diameter. This indicates greater instability in the high-power laser SLM process when a small beam diameter is used.

**Figure 8:**
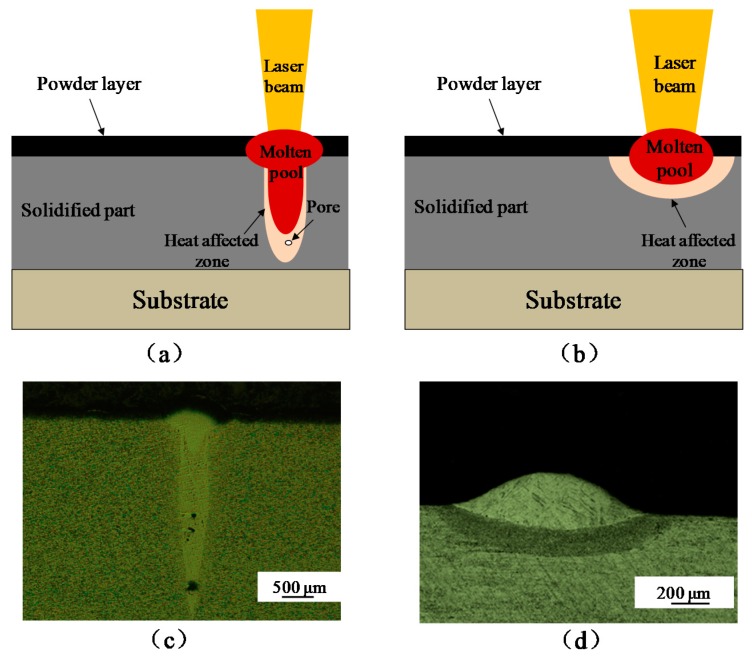
Schematic illustrations and optical micrographs of single-scan tracks formed using different laser beam diameters. Fabrication using a (**a**,**c**) 50-µm beam and (**b**,**d**) a 200-µm beam.

Figure 5, Figure 6 and Figure 7 indicate that the beam diameter significantly influenced the behavior of individual tracks and their geometrical characteristics. Figure 8a shows that using a high laser power while maintaining a small beam diameter gave an intense beam and generated the keyhole. Excessively high laser energy density readily led to improper closure of the keyhole (Figure 8c), which may have been caused by entrapped gases and material evaporation. For example, Ma et al. found that at a higher laser energy input, the residual gas at the bottom of the deep melt pools cannot escape in time during rapid solidification and thus forms pores in the scan track [16]. To avoid instability, the beam diameter must be greater (Figure 8b); increasing the laser spot diameter to 200 µm provided a beam that produced a larger and shallower melt pool. This ensured complete melting of the powder and did not produce a deeper bonding area, as expected (Figure 8d).

In summary, a large-diameter laser seems to be more suited to high laser power SLM technology, especially for thick-layer thicknesses. The surface morphology and cross-sectional characteristics of single-scan tracks suggest a suitable scanning speed of ca. 50 mm/s. These optimal process parameters should produce SLM parts with the desired densities and properties.

### 3.2. Multilayer Fabrication

The effect of beam diameter on densities, microstructures and mechanical properties was investigated in detail. Multilayer blocks were fabricated using different beam diameters at scanning speeds of 25, 50, 75, 100, 125 and 150 mm/s. The hatch spacing was set at 0.7-times the track width. The multilayer test results are shown in Figure 3b, where it is evident that the geometry and dimensional accuracy of the produced parts are poor. Notably, the lower right corner of each sample was sunken. This is because this location was the starting and finishing position of each layer. The long residence and delay closure times resulted in significant overheating of the liquid. The huge recoil pressure acting on the surface of the overheated liquid then caused the scan track to evaporate and collapse.

#### 3.2.1. Densification and Microstructure

**Figure 9:**
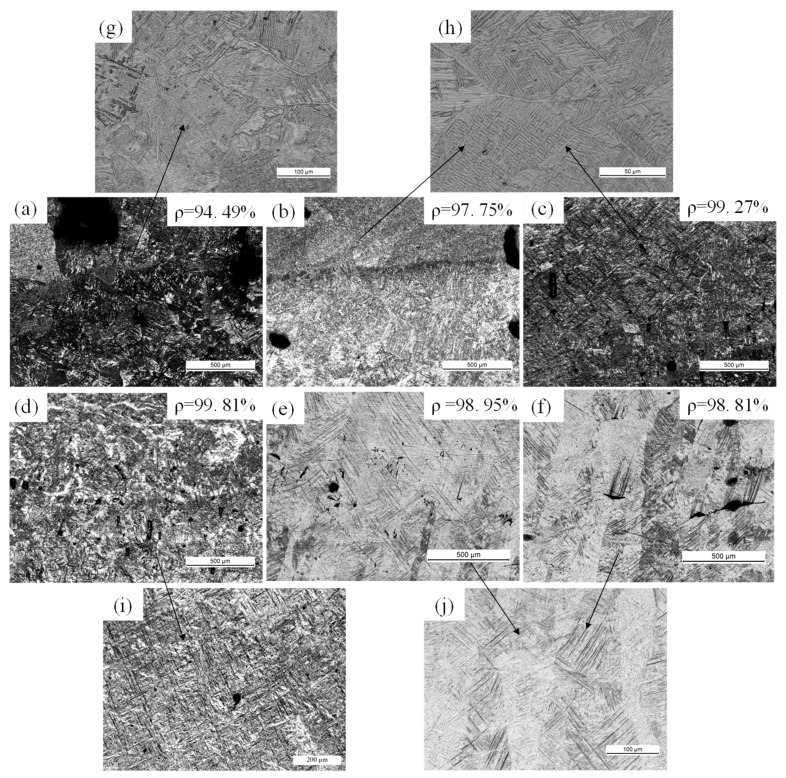
Microstructure and density of selective laser melting (SLM) Ti-6Al-4V samples made at different scanning speeds and hatch spacings using the 50-µm-diameter laser beam. (**a**) v = 25 mm/s; (**b**) v = 50 mm/s; (**c**) v = 75 mm/s; (**d**) v = 100 mm/s; (**e**) v = 125 mm/s; (**f**) v = 150 mm/s. High-magnification optical micrographs showing the (**g**,**h**) Widmanstätten and (**i**,**j**) martensite microstructures.

Figure 9 shows the measured density and microstructure results for the samples fabricated using the 50 µm laser. The density ranged from 94.49% to 99.81% of the theoretical value, which indicated the presence of defects (Figure 9a–f). At a scanning speed of 25 mm/s, the density was 94.49% and large macropores were clearly visible (Figure 9a). The highest density of 99.81% occurred at 100 mm/s (Figure 9d). For a 200-μm-thick layer, a higher energy density is needed to melt the powder layer, but increasing the laser power to 400 W while maintaining a narrow 50 μm beam leads to excessively high laser energy density. This in turn results in higher material evaporation and the formation of holes in the samples. This is clearly an unacceptable SLM process.

The microstructure of Ti-6Al-4V typically consists of a mixture of α and β phases at room temperature [4,24]. Figure 9g–j shows two different kinds of typical microstructures, i.e., the Widmanstätten (Figure 9g,h) and martensite microstructures (Figure 9i,j). The β martensite phase transforms into the α form as the melt cools. The microstructural evolution of Ti-6Al-4V primarily depends on the cooling rate [25]. The transformation of Ti-6Al-4V into the needle-like martensite (α’) occurs at high cooling rates (greater than 410 °C/s) while the lamellar Widmanstätten (α) microstructure forms at intermediate cooling rates (between 20 and 410 °C/s) [26,27]. Generally, the SLM process has high cooling rates that exceed the critical cooling rate of 410 °C/s, which ensures the transformation to martensite α’ [21]. However, SLM of a thick layer at high laser power leads to a high energy density, which keeps the scan tracks in a liquid state for a prolonged time. This reduces the cooling rate. Therefore, at scanning speeds ranging from 25 to 100 mm/s (Figure 9a–d), the high energy input generates the Widmanstätten structure. Figure 9g–h shows that the α lamellae are arranged in a basket-weave structure with different sizes and orientations. With increasing scanning speed, the energy input decreases, resulting in the generation of columnar β grains that consist primarily of acicular martensite (α’) (Figure 9i,j).

**Figure 10:**
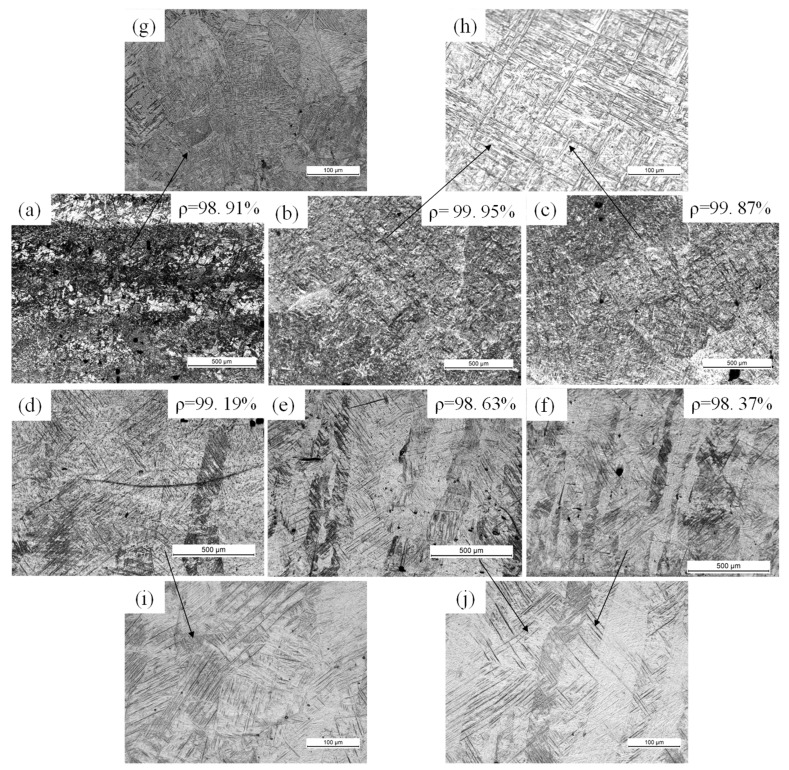
Microstructure and density of SLM Ti-6Al-4V samples made at different scanning speeds and hatch spacings using the 200-µm-diameter laser beam. (**a**) v = 25 mm/s; (**b**) v = 50 mm/s; (**c**) v = 75 mm/s; (**d**) v = 100 mm/s; (**e**) v = 125 mm/s; (**f**) v = 150 mm/s. High-magnification optical micrographs showing the (**g**) Widmanstätten and (**h**,**j**) martensite microstructures.

**Figure 11:**
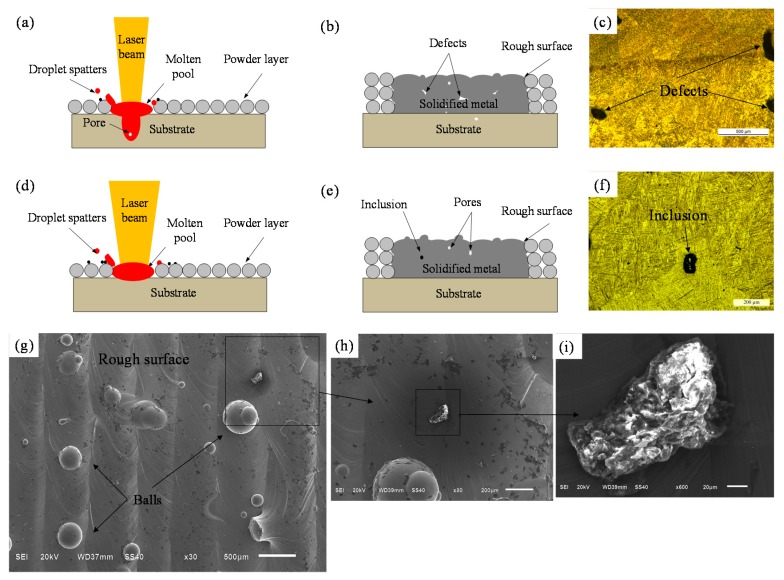
Mechanisms of defect formation for the SLM process using (**a**–**c**) small (50 µm) and (**d**–**f**) large (200 µm) diameter laser beams; (**g**–**i**) Scanning electron micrographs showing the top rough surface structure of the samples.

In contrast, the overall quality of the cross-sectional morphologies of samples made using the large 200-µm-diameter laser (Figure 10) was better than that of samples made with the smaller 50-µm-diameter laser (Figure 9). The density of the samples increased from 98.37–99.95% of the theoretical value as the scanning speed increased from 25 to 150 mm/s. At 25 mm/s, the over-melting zone and large holes were evident in the sample due to the excessive energy input (Figure 10a). No over-melting zone was observed as the speed increased, and the maximum density occurred at a scanning speed of 50 mm/s (Figure 10b). These results are consistent with the single-scan track results (Figure 4 and Figure 6). Hence, SLM processing of thick layers with high laser power can provide high-density metal parts, which can reach 99.95% of the theoretical value. Additionally, increasing the beam diameter could increase the density in a high-power laser SLM process for the Ti-6Al-4V alloy. Figure 11 shows the microstructure of SLM Ti-6Al-4V samples made using the 200-µm-diameter laser. The Widmanstätten and martensite microstructures are clearly observed in the SEM images (Figure 11g–j). At a lower scanning speed of 25 mm/s, the microstructure of the SLM sample was completely Widmanstätten (Figure 10g). At 50 and 75 mm/s, the microstructure consisted of a mixture of Widmanstätten and martensite (Figure 10h). However, at 100 to 150 mm/s, acicular martensite dominated and filled the columnar β grains (Figure 10h–j). The amount of martensite increased with increasing scanning speed because the morphology and size of the microstructures are related to the cooling rate in the SLM process [28].

Forming efficiency is defined as the product of the parameters of layer thickness (δ), scanning speed (v) and hatch spacing (s). For a density greater than 99.9% of the theoretical value, the forming efficiency ranged from 6.3 to 9 mm^3^/s (Φ_D_ = 200 µm: δ = 200 μm, v = 50 mm/s, s = 630 μm; maximum density of 99.95%), which is 2–8 times greater than that obtained with commonly used processing parameters [10,19,26,29].

#### 3.2.2. Mechanisms of Defect Formation

Full density could not be achieved using a high laser power of 400 W and a thick layer of 200 µm. The density of all of the samples made using both the small (50 μm) and large (200 μm) beam diameter ranged from 94.49% to 99.95% (Figure 9 and Figure 10). However, the larger beam diameter generally resulted in higher-density samples.

An understanding of the mechanisms underlying defect formation is needed. Schematic illustrations of this process are given in Figure 11. The spatter phenomenon occurred with both small and large beam diameters (Figure 11a,d). This is because the laser beam profile was Gaussian shaped and the energy intensity changed with position in the laser beam. The intensity was greatest in the middle position and then decreased outwards (Figure 2). Therefore, the region of the Gaussian laser beam that effectively melted the material was primarily located at the center of the beam. When the beam diameter was small, the intensity at the center and outer regions changed only slightly. However, high laser power led to focused and high laser intensity when the keyhole was easily generated (Figure 11a). When the beam diameter is large, the energy input should be increased to allow the energy intensity of the outer regions of the Gaussian beam to fully melt the thick powder layer. However, when this occurs, the energy intensity experienced at the center of the Gaussian beam becomes excessive and causes the molten material at the center of the melt pool to splash and evaporate [14]. Inclusions and unmelted defects were evident in samples made with the large beam diameter of 200 µm (Figure 11e,f). Additionally, the small-diameter laser was more likely to produce more and larger defects (Figure 11b,c) due to more serious spattering and evaporation.

High laser power and layer thickness strongly affected the surface roughness during the SLM process. In the experiments, a large number of balls and slag inclusions were spattered during the SLM process and all of the samples consequently had relatively rough surfaces (Figure 11g–i). This occurred mainly because a high layer thickness needs high laser power and high energy input to fully melt the powder. However, this increases the intensity of the laser energy, which in turn increases the evaporation rate and the spattering and causes a large recoil pressure on the surface of the overheated liquid, resulting in an uneven surface [7]. Further research is warranted to identify ways to eliminate process instabilities and improve forming precision.

#### 3.2.3. Tensile Properties

**Figure 12:**
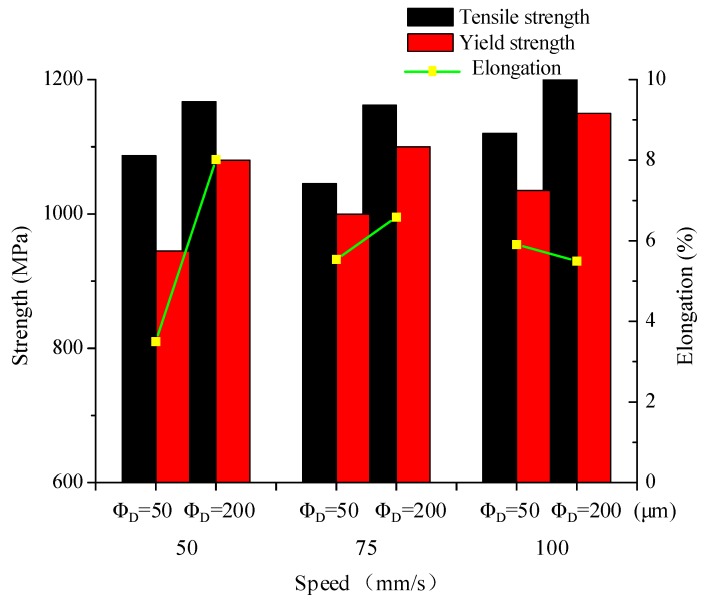
Tensile properties of samples produced by SLM using different laser beam diameters.

The tensile test results of the SLM samples made using different laser beam diameters are summarized in Figure 12. Samples made with the larger diameter (200 µm) laser had better tensile properties than those made with the small (50 µm) diameter laser. With the 200 µm beam, the ultimate tensile strength (UTS) ranged from 1147 to 1200 MPa, yield strength from 1090 to 1150 MPa and elongation from 5.51% to 8.02%. However, with the 50-µm laser, the UTS ranged from 997 to 1100 MPa, yield strength from 945 to 1035 MPa and elongation from 3.52% to 5.91%. The optimal tensile properties using the small beam diameter were obtained at a scanning speed of 100 mm/s when the yield strength, UTS and elongation were 1035 MPa, 1100 MPa and 5.91%, respectively. Samples fabricated at the same scanning speed but using the large beam diameter exhibited higher yield strength of 1090 MPa and UTS of 1168 MPa, and the elongation of 5.51%. However, the optimal elongation of 8.02% was attained at a scanning speed of 50 mm/s. Notably, these values do not meet those specified by the ASTM B348-13 standard for Ti-6Al-4V (Grade 5), as shown in Table 2.

These findings are primarily attributed to density and microstructural differences (Figure 9 and Figure 10). The mechanical properties improved with increasing density. Therefore, the lower densities obtained using the small-diameter laser resulted in materials having lower tensile strength and ductility. Large amounts of acicular martensite (α’) provide high mechanical strength but low ductility [30,31], while a microstructure that is composed mostly of the lamellar martensite (α) phase results in a material having high ductility but low mechanical strength. Accordingly, the yield strength and UTS were higher at the higher scanning speed of 100 mm/s (Figure 10d) than at 50 mm/s, although the density was slightly lower (Figure 10b).

**Figure 13:**
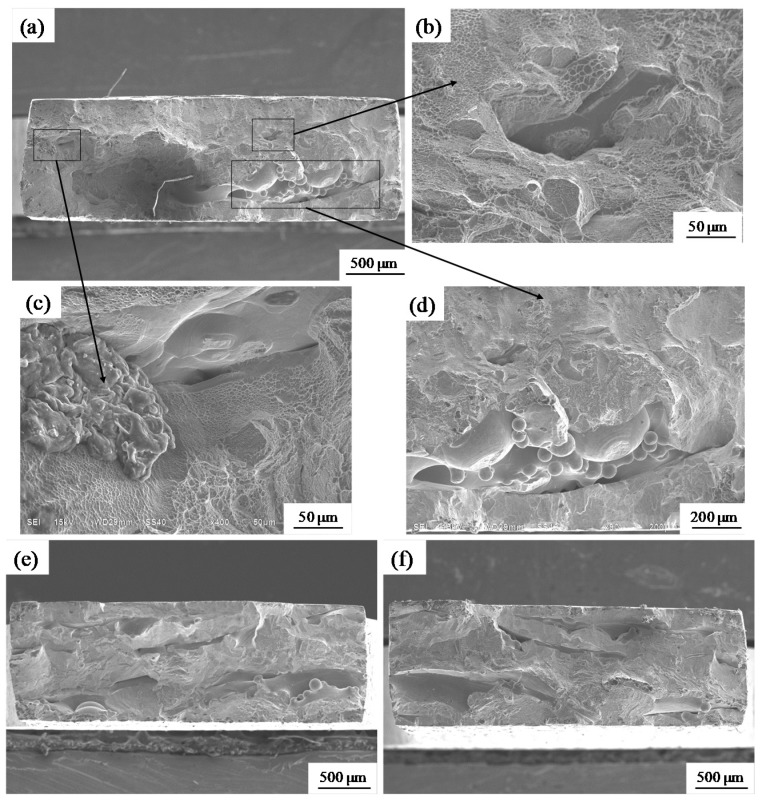
Scanning electron micrographs of tensile fracture surfaces of SLM-produced specimens. (**a**) Overall view of the tensile specimen (Φ_D_ = 50 μm, v = 75 mm/s); (**b**–**d**) Magnified views of the boxed region in image (**a**); (**e**) Overall view of the tensile specimen (Φ_D_ = 200 μm, v = 75 mm/s); (**f**) Overall view of the tensile specimen (Φ_D_ = 200 μm, v = 100 mm/s).

**Figure 14:**
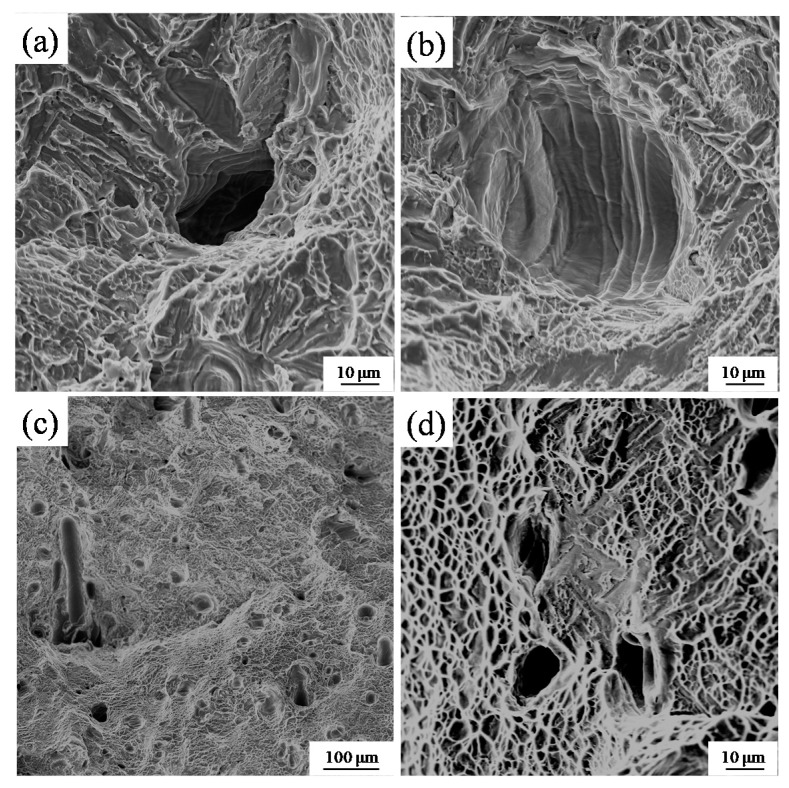
Scanning electron micrographs showing the defect morphologies and rupture features. (**a**) Holes; (**b**) brittle fracture; (**c**) slag inclusions; (**d**) tough fracture.

Representative fractographs of the tensile fracture surface of SLM-produced Ti-6Al-4V test specimens are shown in Figure 13. The fracture characteristics were a mixture of tough and brittle [20]. Carbides are evident at the left side of the fracture (Figure 13c) and spherulized granules at the lower right corner of the fracture (Figure 13d) relate to brittle fracture. These metal inclusions may be over-melted particles, oxidized particles or particles solidified after splashing. Such defects greatly reduce the overall mechanical performance of a specimen. In a defect-free part, the fracture appears as a torn edge (Figure 13b), which is associated with ductile fracture; there are many tiny dimples beside the torn edge. There were more torn edges when the beam diameter was large (Figure 13e,f), but a small number of defects also led to brittle fracture, resulting in reduced performance. High-magnification SEM micrographs clearly revealed the defect morphologies (Figure 14). Holes (Figure 14a) and slag inclusions (Figure 14c) were the main defects in the experimental SLM samples, and typical brittle-fracture features, such as cleaved surfaces and torn ridges, were evident around these defects (Figure 14b). These defects are responsible for the lower density and poor mechanical properties noted above. The fracture surfaces of high-density samples fabricated using the large beam diameter were replete with small dimple-like structures (Figure 14d), which are indicative of ductile rupture.

## 4. Conclusions

This study conducted a thorough investigation of the effects of different laser beam diameters (50 μm and 200 μm) on the density, microstructure and mechanical properties of the Ti-6Al-4V alloy. The main findings are as follows:

1. A series of single-track experiments demonstrated the difficulty in forming smooth and defect-free scan tracks using the small-diameter beam. Increasing the beam diameter mitigated process instability and provided a more stable and uniform melt pool.
2. Defects were identified in all of the fabricated samples. The small-diameter laser was more prone to producing an over-melted zone and large holes due to excessive energy input. Increasing the beam diameter enhanced the density in high-power SLM process. Samples made using the small-diameter (50 µm) laser had densities that ranged from 94.49% to 99.81%, while those fabricated using the large-diameter laser (200 µm) laser had densities that ranged from 98.37% to 99.95%.
3. A large number of balls and slag inclusions were spattered during the SLM process, which resulted in relatively rough surfaces for all of the samples. Additionally, the small-diameter laser was more likely to produce more and larger defects.
4. With the large beam diameter of 200 µm, the optimal yield strength, UTS and elongation were 1150 MPa, 1200 MPa and 8.02%, respectively. The corresponding values with the small beam diameter of 50 µm were 1035 MPa, 1100 MPa and 5.91%. Overall, the large-diameter laser is more suitable for high-power SLM technology, especially for thick layers.

## Figures and Tables

**Table 1:** The chemical composition of Ti-6Al-4V powder.

Element	Al	V	Fe	O	C	N	H	Ti
wt.%	5.8	3.8	0.3	0.15	0.02	0.05	0.03	Balance

**Table 2:** Comparison of the mechanical properties.

References	Yield Strength (MPa)	Ultimate Tensile Stress (MPa)	Elongation (%)
ASTM B348-13	828	895	10
Φ_D_ = 50 µm In this study	1035	1100	5.91
Φ_D_ = 200 µm In this study	1150	1200	8.02

## References

[1] Kumar S. (2014). Selective Laser Sintering/Melting. Compr. Mater. Process..

[2] Gasser A., Backes G., Kelbassa I., Weisheit A., Wissenbach K. (2010). Laser additive manufacturing. Laser Tech. J..

[3] Bremen S., Meiners W., Diatlov A. (2012). Selective Laser Melting. Laser Tech. J..

[4] Fousova M., Vojtech D., Doubrava K., Daniel M., Lin C.F. (2018). Influence of Inherent Surface and Internal Defects on Mechanical Properties of Additively Manufactured Ti6Al4V Alloy: Comparison between Selective Laser Melting and Electron Beam Melting. Materials.

[5] Gu D., Wang H., Dai D., Yuan P., Meiners W., Poprawe R. (2015). Rapid fabrication of Al-based bulk-form nanocomposites with novel reinforcement and enhanced performance by selective laser melting. Scr. Mater..

[6] Baitimerov R., Lykov P., Zherebtsov D., Radionova L., Shultc A., Prashanth K.G. (2018). Influence of Powder Characteristics on Processability of AlSi12 Alloy Fabricated by Selective Laser Melting. Materials.

[7] Qiu C., Panwisawas C., Ward M., Basoalto H.C., Brooks J.W., Attallah M.M. (2015). On the role of melt flow into the surface structure and porosity development during selective laser melting. Acta Mater..

[8] Attar H., Bönisch M., Calin M., Zhang L.-C., Scudino S., Eckert J. (2014). Selective laser melting of in situ titanium–titanium boride composites: Processing, microstructure and mechanical properties. Acta Mater..

[9] Han X., Zhu H., Nie X., Wang G., Zeng X. (2018). Investigation on Selective Laser Melting AlSi10Mg Cellular Lattice Strut: Molten Pool Morphology, Surface Roughness and Dimensional Accuracy. Materials.

[10] Shi X., Ma S., Liu C., Chen C., Wu Q., Chen X., Lu J. (2016). Performance of High Layer Thickness in Selective Laser Melting of Ti6Al4V. Materials.

[11] Koutny D., Palousek D., Pantelejev L., Hoeller C., Pichler R., Tesicky L., Kaiser J. (2018). Influence of Scanning Strategies on Processing of Aluminum Alloy EN AW 2618 Using Selective Laser Melting. Materials.

[12] Kuo Y.L., Nagahari T., Kakehi K. (2018). The Effect of Post-Processes on the Microstructure and Creep Properties of Alloy718 Built Up by Selective Laser Melting. Materials.

[13] Shi X., Ma S., Liu C., Wu Q. (2017). Parameter optimization for Ti-47Al-2Cr-2Nb in selective laser melting based on geometric characteristics of single scan tracks. Opt. Laser Technol..

[14] Schleifenbaum H., Meiners W., Wissenbach K., Hinke C. (2010). Individualized production by means of high power Selective Laser Melting. CIRP J. Manuf. Sci. Technol..

[15] Vastola G., Zhang G., Pei Q.X., Zhang Y.W. (2015). Modeling and control of remelting in high-energy beam additive manufacturing. Addit. Manuf..

[16] Ma M., Wang Z., Gao M., Zeng X. (2014). Layer thickness dependence of performance in high-power selective laser melting of 1Cr18Ni9Ti stainless steel. J. Mater. Process. Technol..

[17] Buchbinder D., Meiners W., Pirch N., Wissenbach K., Schrage J. (2014). Investigation on reducing distortion by preheating during manufacture of aluminum components using selective laser melting. J. Laser Appl..

[18] (2013). Astm F136-02a, Standard Specification For Wrought Titanium-6aluminum-4vanadium Eli (Extra Low Interstitial) Alloy For Surgical Implant Applications.

[19] Campanelli S.L., Contuzzi N., Ludovico A.D., Caiazzo F., Cardaropoli F., Sergi V. (2014). Manufacturing and characterization of Ti6Al4V lattice components manufactured by selective laser melting. Materials.

[20] Li T., Jiang F., Olevsky E.A., Vecchio K.S., Meyers M.A. (2007). Damage evolution in Ti6Al4V–Al3Ti metal-intermetallic laminate composites. Mater. Sci. Eng. A.

[21] Kelly S.M., Kampe S.L. (2004). Microstructural evolution in laser-deposited multilayer Ti-6Al-4V builds: Part II. Thermal modeling. Metall. Mater. Trans. A.

[22] Yadroitsev I., Gusarov A., Yadroitsava I., Smurov I. (2010). Single track formation in selective laser melting of metal powders. J. Mater. Process. Technol..

[23] Yadroitsev I., Bertrand P., Antonenkova G., Grigoriev S., Smurov I. (2013). Use of track/layer morphology to develop functional parts by selective laser melting. J. Laser Appl..

[24] Juechter V., Scharowsky T., Singer R.F., Körner C. (2014). Processing window and evaporation phenomena for Ti–6Al–4V produced by selective electron beam melting. Acta Mater..

[25] Simonelli M. (2014). Microstructure Evolution and Mechanical Properties of Selective Laser Melted Ti-6Al-4V.

[26] Thijs L., Verhaeghe F., Craeghs T., Humbeeck J.V., Kruth J.-P. (2010). A study of the microstructural evolution during selective laser melting of Ti–6Al–4V. Acta Mater..

[27] Shi X., Ma S., Liu C., Wu Q., Lu J., Liu Y., Shi W. (2017). Selective laser melting-wire arc additive manufacturing hybrid fabrication of Ti-6Al-4V alloy: Microstructure and mechanical properties. Mater. Sci. Eng. A.

[28] Baufeld B., Van der Biest O., Gault R., Ridgway K. (2011). Manufacturing Ti-6Al-4V Components by Shaped Metal Deposition: Microstructure and Mechanical Properties. IOP Conf. Ser. Mater. Sci. Eng..

[29] Facchini L., Magalini E., Robotti P., Molinari A., Höges S., Wissenbach K. (2010). Ductility of a Ti-6Al-4V alloy produced by selective laser melting of prealloyed powders. Rapid Prototyp. J..

[30] Romano J., Ladani L., Razmi J., Sadowski M. (2015). Temperature distribution and melt geometry in laser and electron-beam melting processes—A comparison among common materials. Addit. Manuf..

[31] Antonysamy A.A., Meyer J., Prangnell P.B. (2013). Effect of build geometry on the β-grain structure and texture in additive manufacture of Ti6Al4V by selective electron beam melting. Mater. Charact..

